# Supplementary material for: The cost of the plunge: the impact and cost of a cessation of PEPFAR-supported services in South Africa
Source: AIDS. 2025 Jun 20;39(10):1476–80. doi: 10.1097/QAD.0000000000004272 (PMC12262123; doi:10.1097/QAD.0000000000004272)
Supplement: Supplementary file 1 [file aids-39-1476-s001.docx]

| **Supplementary Table 1: Expenditure on South African HIV programme in 2023 by funder** [1] | | | | | | | |
| --- | --- | --- | --- | --- | --- | --- | --- |
| **Intervention** | **Total amounts (2023 USD)** | | | | **Percentage of total** | | |
|  | **SAG** | **PEPFAR** | **Global Fund** | **Total** | **DOH** | **PEPFAR** | **GF** |
| **Treatment, care and support** | **$1,130,133,344** | **$192,378,465** | **$20,328,191** | **$1,342,840,000** | **84%** | **14%** | **2%** |
| Antiretroviral treatment | $456,610,142 | - | $20,050,603 | **$476,660,745** | 96% | - | 4% |
| HIV-related laboratory monitoring | $340,414,787 | $623,878 | $8,959 | **$341,047,624** | 100% | - | - |
| Opportunistic infections (OI) prophylaxis and treatment | $38,295 | - | $268,629 | **$306,924** | 12% | - | 88% |
| Programmatic activities for treatment, care and support | $298,150,385 | $191,754,587 | - | **$489,904,972** | 61% | 39% | - |
| Support and retention | $34,919,735 | - | - | **$34,919,735** | 100% | - | - |
| Palliative care | $539,607 | - | - | **$539,607** | 100% | - | - |
| **Testing** | **$52,942,909** | **$3,393,131** | **-** | **$56,336,040** | **94%** | **6%** | **-** |
| HIV testing and counselling | $52,942,909 | $3,393,131 | - | **$56,336,040** | 94% | 6% | - |
| **Prevention** | **$62,192,180** | **$68,330,843** | **$6,154,270** | **$136,677,291** | **46%** | **50%** | **5%** |
| MMC | $32,513,708 | $26,822,843 | - | **$59,336,551** | 55% | 45% | - |
| Condoms | $19,711,908 | - | $2,263 | **$19,714,171** | 100% | - | 0,01% |
| PMTCT | $6,117,827 | - | - | **$6,117,827** | 100% | - | - |
| Prevention and promotion of testing and linkage for KP | $607,077 | $6,326,613 | $2,537,434 | $9,471,123 | 6% | 67% | 27% |
| PEP | $937,335 | - | - | **$937,335** | 100% | - | - |
| PrEP (AGYW) | - | $15,496,310 | - | **$15,496,310** | - | 100%^[[1]](#footnote-1)^ | - |
| PrEP (general population) | $265,712 | $494,740 | $148,563 | **$909,015** | 29% | 54% | 16% |
| PrEP (MSM) | - | $773,571 | - | **$773,571** | - | 100% | - |
| PrEP (prisoners) | - | $389,148 | - | **$389,148** | - | 100% | - |
| PrEP (SW) | - | $196,157 | - | **$196,157** | - | 100% | - |
| PrEP (PWID) | - | $49,901 | - | **$49,901** | - | 100% | - |
| Other prevention | $1,499,006 | $17,781,560 | $3,466,010 | **$22,746,575** | 7% | 78% | 15% |
| **Other programmes** | **$925,526** | **$-** | **$2,744,474** | **$3,670,000** | **25%** | **-** | **75%** |
| Social and behaviour change (SBC) programmes | - | - | $2,744,474 | **$2,744,474** | - | - | 100% |
| Workplace programmes | $925,526 | - | - | **$925,526** | 100% | - | - |
| **Total** | **$1,250,594,095** | **$272,764,187** | **$35,888,383** | **$1,559,246,664** | **80%** | **17%** | **2%** |
| System strengthening and enablers | $170,059,771 | $115,233,016 | $18,377,691 | $303,670,478 | 56% | 38% | 6% |
| **Overall total** | **$1,420,653,866** | **$387,997,202** | **$54,266,074** | **$1,862,917,142** | **76%** | **21%** | **3%** |

1. Note that even though PEPFAR funded 100% of PrEP services for specific population groups in 2023, the last year that complete expenditure data has been reported for, this changed to 20% in 2024, with the SAG taking over the majority of PrEP provision, according to SAG data. In our minimum scenario, we therefore assume a reduction by 20% in PrEP coverage in these population groups. [↑](#footnote-ref-1)
