# Supplementary material for: The cost of the plunge: the impact and cost of a cessation of PEPFAR-supported services in South Africa
Source: AIDS. 2025 Jun 20;39(10):1476–80. doi: 10.1097/QAD.0000000000004272 (PMC12262123; doi:10.1097/QAD.0000000000004272)
Supplement: Supplementary file 2 [file aids-39-1476-s002.docx]

**Supplementary Table 2A: Cost effectiveness of preserving interventions over baseline of defunding HIV programme by 21% (2025-2029)**

| **Scenario** | **HIV infections averted** | **AIDS deaths averted** | **Life years saved** | **Additional cost [mil’ 2024 USD]** | **Cost (USD)/HIV infection averted** | **Cost (USD)/ death averted** | **Cost (USD)/ life year saved** |
| --- | --- | --- | --- | --- | --- | --- | --- |
| **Keep: PrEP at current coverage**  Reduce: ART by 14%, HTS by 6%, MMC by 45% | 144,816 | 55,721 | 1,274,912 | 542 | 3,740 | 9,719 | 425 |
| **Keep: ART at current coverage**  Reduce: HTS by 6%, MMC by 45%, PrEP in KPs by 20% | 2,162 | 331 | 7,722 | 24 | 11,271 | 73,611 | 3,155 |
| **Keep: HTS at current coverage**  Reduce: ART by 14%, MMC by 45%, PrEP in KPs by 20% | 987 | 2 | 56 | 2 | 1,992 | 965,563 | 35,293 |
| **Keep: MMC at current coverage**  Reduce: ART by 14%, HTS by 6%, PrEP in KPs by 20% | 1,958 | 4 | 104 | 49 | 25,021 | 12,501,979 | 469,177 |
