# Supplementary material for: The cost of the plunge: the impact and cost of a cessation of PEPFAR-supported services in South Africa
Source: AIDS. 2025 Jun 20;39(10):1476–80. doi: 10.1097/QAD.0000000000004272 (PMC12262123; doi:10.1097/QAD.0000000000004272)
Supplement: Supplementary file 3 [file aids-39-1476-s003.docx]

**Supplementary Table 2B: Cost effectiveness of preserving interventions over baseline of defunding HIV programme by 21% (2025-2044)**

| **Scenario** | **HIV infections averted** | **AIDS deaths averted** | **Life years saved** | **Additional cost [mil’ 2024 USD]** | **Cost (USD)/HIV infection averted** | **Cost (USD)/ death averted** | **Cost (USD)/ life year saved** |
| --- | --- | --- | --- | --- | --- | --- | --- |
| **Keep: PrEP at current coverage**  Reduce: ART by 14%, HTS by 6%, MMC by 45% | 1,144 | 158 | 3,937 | -6 | Cost-saving | | |
| **Keep: ART at current coverage**  Reduce: HTS by 6%, MMC by 45%, PrEP in KPs by 20% | 266,562 | 125,047 | 2,899,090 | 187 | 703 | 1,499 | 65 |
| **Keep: HTS at current coverage**  Reduce: ART by 14%, MMC by 45%, PrEP in KPs by 20% | 7,765 | 2,146 | 50,810 | 7 | 918 | 3,322 | 140 |
| **Keep: MMC at current coverage**  Reduce: ART by 14%, HTS by 6%, PrEP in KPs by 20% | 38,686 | 1,499 | 38,216 | 27 | 698 | 18,007 | 706 |
